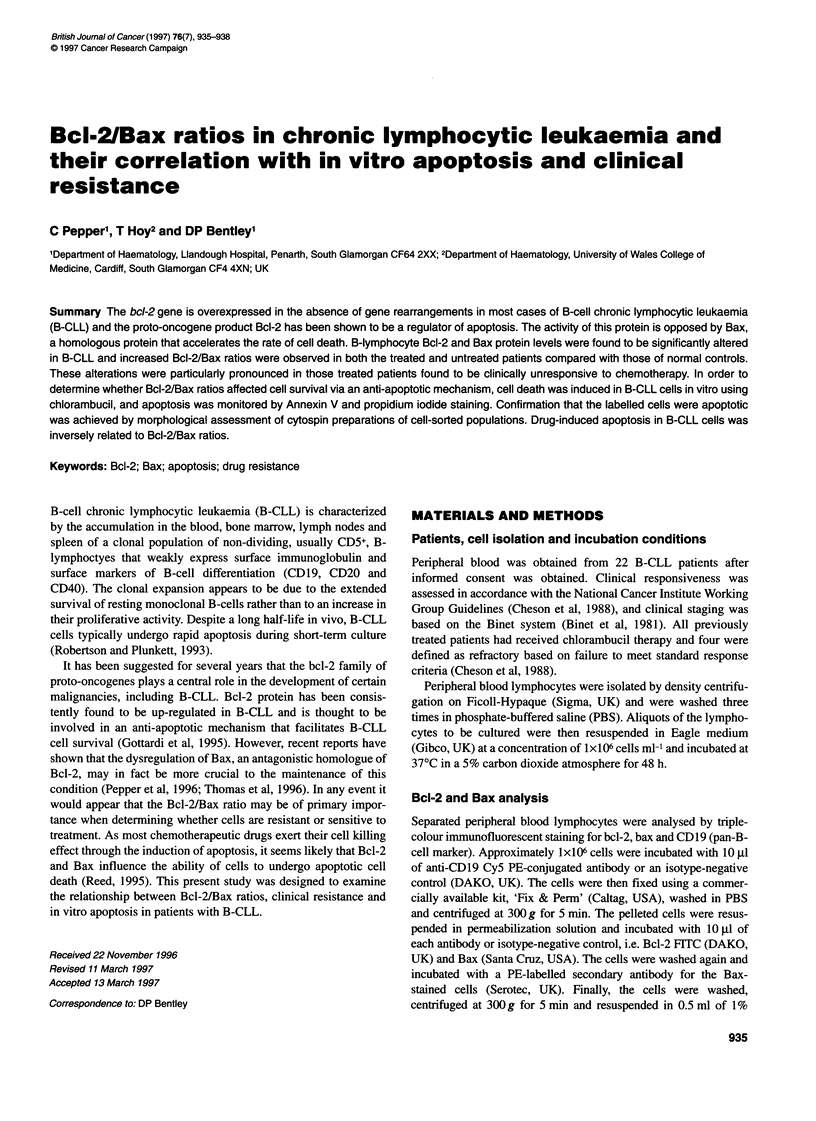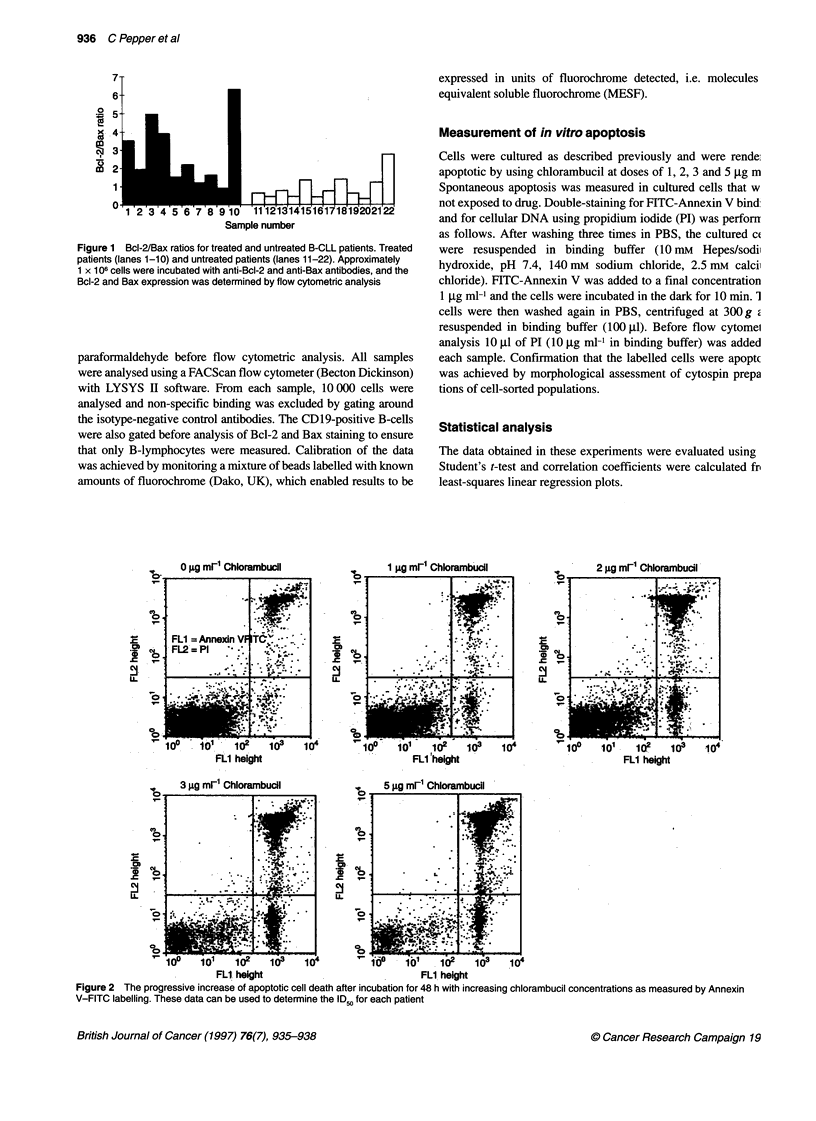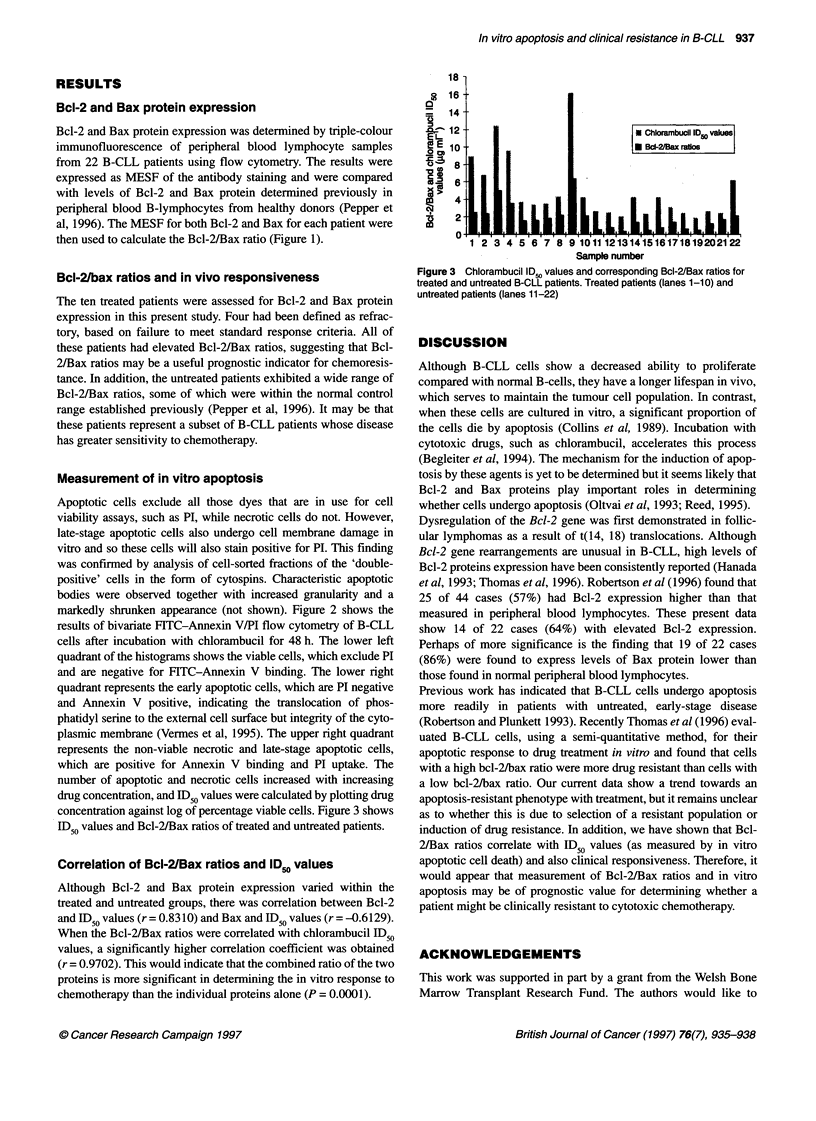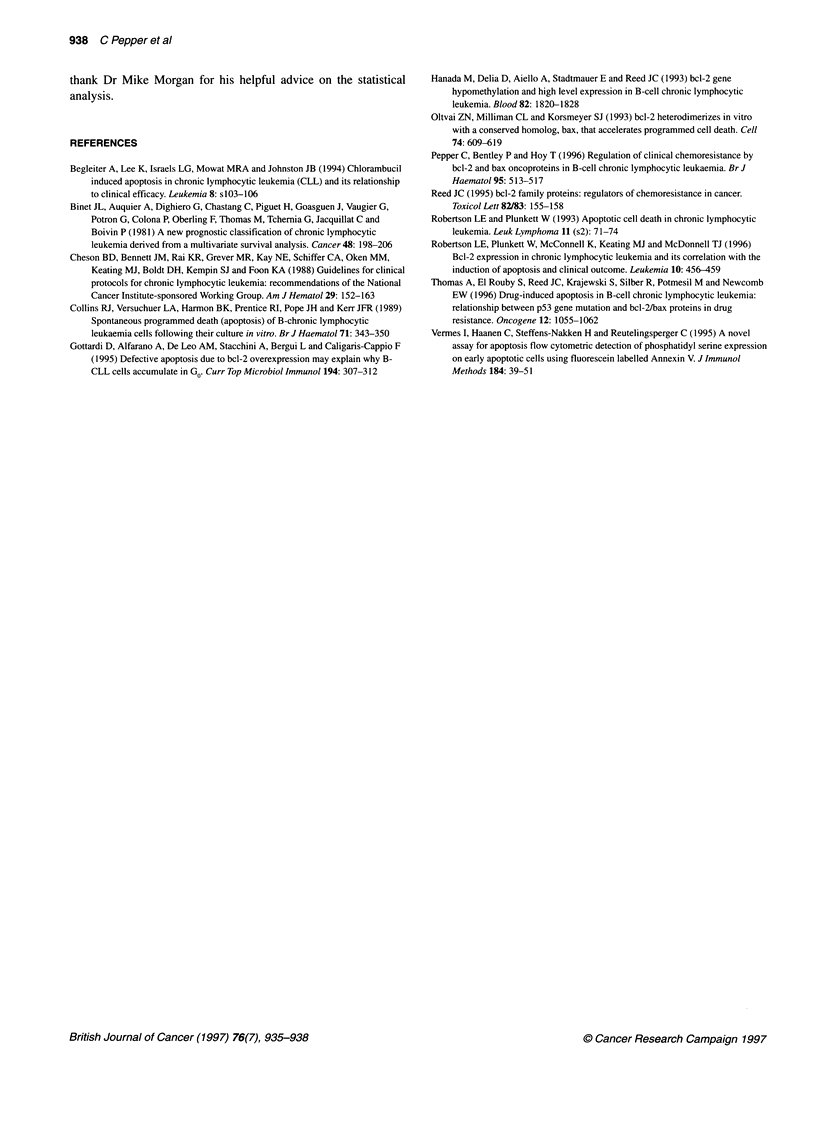# Bcl-2/Bax ratios in chronic lymphocytic leukaemia and their correlation with in vitro apoptosis and clinical resistance.

**DOI:** 10.1038/bjc.1997.487

**Published:** 1997

**Authors:** C. Pepper, T. Hoy, D. P. Bentley

**Affiliations:** Department of Haematology, Llandough Hospital, Penarth, South Glamorgan, UK.

## Abstract

The bcl-2 gene is overexpressed in the absence of gene rearrangements in most cases of B-cell chronic lymphocytic leukaemia (B-CLL) and the proto-oncogene product Bcl-2 has been shown to be a regulator of apoptosis. The activity of this protein is opposed by Bax, a homologous protein that accelerates the rate of cell death. B-lymphocyte Bcl-2 and Bax protein levels were found to be significantly altered in B-CLL and increased Bcl-2/Bax ratios were observed in both the treated and untreated patients compared with those of normal controls. These alterations were particularly pronounced in those treated patients found to be clinically unresponsive to chemotherapy. In order to determine whether Bcl-2/Bax ratios affected cell survival via an anti-apoptotic mechanism, cell death was induced in B-CLL cells in vitro using chlorambucil, and apoptosis was monitored by Annexin V and propidium iodide staining. Confirmation that the labelled cells were apoptotic was achieved by morphological assessment of cytospin preparations of cell-sorted populations. Drug-induced apoptosis in B-CLL cells was inversely related to Bcl-2/Bax ratios.


British Journal of Cancer (1997) 76(7), 935–938
© 1997 Cancer Research Campaign

### Bcl-2/Bax ratios in chronic lymphocytic leukaemia and their correlation with in vitro apoptosis and clinical resistance

**C Pepper[1], T Hoy[2] and DP Bentley[1]**

[1]Department of Haematology, Llandough Hospital, Penarth, South Glamorgan CF64 2XX; [2]Department of Haematology, University of Wales College of Medicine, Cardiff, South Glamorgan CF4 4XN; UK

**Summary** The *bcl-2* gene is overexpressed in the absence of gene rearrangements in most cases of B-cell chronic lymphocytic leukaemia (B-CLL) and the proto-oncogene product Bcl-2 has been shown to be a regulator of apoptosis. The activity of this protein is opposed by Bax, a homologous protein that accelerates the rate of cell death. B-lymphocyte Bcl-2 and Bax protein levels were found to be significantly altered in B-CLL and increased Bcl-2/Bax ratios were observed in both the treated and untreated patients compared with those of normal controls. These alterations were particularly pronounced in those treated patients found to be clinically unresponsive to chemotherapy. In order to determine whether Bcl-2/Bax ratios affected cell survival via an anti-apoptotic mechanism, cell death was induced in B-CLL cells in vitro using chlorambucil, and apoptosis was monitored by Annexin V and propidium iodide staining. Confirmation that the labelled cells were apoptotic was achieved by morphological assessment of cytospin preparations of cell-sorted populations. Drug-induced apoptosis in B-CLL cells was inversely related to Bcl-2/Bax ratios.

**Keywords:** Bcl-2; Bax; apoptosis; drug resistance

B-cell chronic lymphocytic leukaemia (B-CLL) is characterized by the accumulation in the blood, bone marrow, lymph nodes and spleen of a clonal population of non-dividing, usually $CD5^+$, B-lymphoctyes that weakly express surface immunoglobulin and surface markers of B-cell differentiation (CD19, CD20 and CD40). The clonal expansion appears to be due to the extended survival of resting monoclonal B-cells rather than to an increase in their proliferative activity. Despite a long half-life in vivo, B-CLL cells typically undergo rapid apoptosis during short-term culture (Robertson and Plunkett, 1993).

It has been suggested for several years that the bcl-2 family of proto-oncogenes plays a central role in the development of certain malignancies, including B-CLL. Bcl-2 protein has been consistently found to be up-regulated in B-CLL and is thought to be involved in an anti-apoptotic mechanism that facilitates B-CLL cell survival (Gottardi et al, 1995). However, recent reports have shown that the dysregulation of Bax, an antagonistic homologue of Bcl-2, may in fact be more crucial to the maintenance of this condition (Pepper et al, 1996; Thomas et al, 1996). In any event it would appear that the Bcl-2/Bax ratio may be of primary importance when determining whether cells are resistant or sensitive to treatment. As most chemotherapeutic drugs exert their cell killing effect through the induction of apoptosis, it seems likely that Bcl-2 and Bax influence the ability of cells to undergo apoptotic cell death (Reed, 1995). This present study was designed to examine the relationship between Bcl-2/Bax ratios, clinical resistance and in vitro apoptosis in patients with B-CLL.

*Received 22 November 1996*
*Revised 11 March 1997*
*Accepted 13 March 1997*

*Correspondence to:* DP Bentley

### MATERIALS AND METHODS

### Patients, cell isolation and incubation conditions

Peripheral blood was obtained from 22 B-CLL patients after informed consent was obtained. Clinical responsiveness was assessed in accordance with the National Cancer Institute Working Group Guidelines (Cheson et al, 1988), and clinical staging was based on the Binet system (Binet et al, 1981). All previously treated patients had received chlorambucil therapy and four were defined as refractory based on failure to meet standard response criteria (Cheson et al, 1988).

Peripheral blood lymphocytes were isolated by density centrifugation on Ficoll-Hypaque (Sigma, UK) and were washed three times in phosphate-buffered saline (PBS). Aliquots of the lymphocytes to be cultured were then resuspended in Eagle medium (Gibco, UK) at a concentration of $1{\times}10^6$ cells $ml^{-1}$ and incubated at 37°C in a 5% carbon dioxide atmosphere for 48 h.

### Bcl-2 and Bax analysis

Separated peripheral blood lymphocytes were analysed by triple-colour immunofluorescent staining for bcl-2, bax and CD19 (pan-B-cell marker). Approximately $1{\times}10^6$ cells were incubated with 10 µl of anti-CD19 Cy5 PE-conjugated antibody or an isotype-negative control (DAKO, UK). The cells were then fixed using a commercially available kit, 'Fix & Perm' (Caltag, USA), washed in PBS and centrifuged at 300 *g* for 5 min. The pelleted cells were resuspended in permeabilization solution and incubated with 10 µl of each antibody or isotype-negative control, i.e. Bcl-2 FITC (DAKO, UK) and Bax (Santa Cruz, USA). The cells were washed again and incubated with a PE-labelled secondary antibody for the Bax-stained cells (Serotec, UK). Finally, the cells were washed, centrifuged at 300 *g* for 5 min and resuspended in 0.5 ml of 1%

**Figure 1** Bcl-2/Bax ratios for treated and untreated B-CLL patients. Treated patients (lanes 1–10) and untreated patients (lanes 11–22). Approximately $1 \times 10^6$ cells were incubated with anti-Bcl-2 and anti-Bax antibodies, and the Bcl-2 and Bax expression was determined by flow cytometric analysis paraformaldehyde before flow cytometric analysis. All samples were analysed using a FACScan flow cytometer (Becton Dickinson) with LYSYS II software. From each sample, 10 000 cells were analysed and non-specific binding was excluded by gating around the isotype-negative control antibodies. The CD19-positive B-cells were also gated before analysis of Bcl-2 and Bax staining to ensure that only B-lymphocytes were measured. Calibration of the data was achieved by monitoring a mixture of beads labelled with known amounts of fluorochrome (Dako, UK), which enabled results to be expressed in units of fluorochrome detected, i.e. molecules equivalent soluble fluorochrome (MESF).

### Measurement of *in vitro* apoptosis

Cells were cultured as described previously and were rende apoptotic by using chlorambucil at doses of 1, 2, 3 and 5 μg m Spontaneous apoptosis was measured in cultured cells that w not exposed to drug. Double-staining for FITC-Annexin V bind and for cellular DNA using propidium iodide (PI) was perforn as follows. After washing three times in PBS, the cultured ce were resuspended in binding buffer (10 mM Hepes/sodi hydroxide, pH 7.4, 140 mM sodium chloride, 2.5 mM calci chloride). FITC-Annexin V was added to a final concentration 1 μg ml$^{-1}$ and the cells were incubated in the dark for 10 min. T cells were then washed again in PBS, centrifuged at 300 ***g*** a resuspended in binding buffer (100 μl). Before flow cytomet analysis 10 μl of PI (10 μg ml$^{-1}$ in binding buffer) was added each sample. Confirmation that the labelled cells were apopto was achieved by morphological assessment of cytospin prepa tions of cell-sorted populations.

### Statistical analysis

The data obtained in these experiments were evaluated using Student's *t*-test and correlation coefficients were calculated fr least-squares linear regression plots.

**Figure 2** The progressive increase of apoptotic cell death after incubation for 48 h with increasing chlorambucil concentrations as measured by Annexin V–FITC labelling. These data can be used to determine the $ID_{50}$ for each patient

*© Cancer Research Campaign 19*

### RESULTS

### Bcl-2 and Bax protein expression

Bcl-2 and Bax protein expression was determined by triple-colour immunofluorescence of peripheral blood lymphocyte samples from 22 B-CLL patients using flow cytometry. The results were expressed as MESF of the antibody staining and were compared with levels of Bcl-2 and Bax protein determined previously in peripheral blood B-lymphocytes from healthy donors (Pepper et al, 1996). The MESF for both Bcl-2 and Bax for each patient were then used to calculate the Bcl-2/Bax ratio (Figure 1).

### Bcl-2/bax ratios and in vivo responsiveness

The ten treated patients were assessed for Bcl-2 and Bax protein expression in this present study. Four had been defined as refractory, based on failure to meet standard response criteria. All of these patients had elevated Bcl-2/Bax ratios, suggesting that Bcl-2/Bax ratios may be a useful prognostic indicator for chemoresistance. In addition, the untreated patients exhibited a wide range of Bcl-2/Bax ratios, some of which were within the normal control range established previously (Pepper et al, 1996). It may be that these patients represent a subset of B-CLL patients whose disease has greater sensitivity to chemotherapy.

### Measurement of in vitro apoptosis

Apoptotic cells exclude all those dyes that are in use for cell viability assays, such as PI, while necrotic cells do not. However, late-stage apoptotic cells also undergo cell membrane damage in vitro and so these cells will also stain positive for PI. This finding was confirmed by analysis of cell-sorted fractions of the 'double-positive' cells in the form of cytospins. Characteristic apoptotic bodies were observed together with increased granularity and a markedly shrunken appearance (not shown). Figure 2 shows the results of bivariate FITC–Annexin V/PI flow cytometry of B-CLL cells after incubation with chlorambucil for 48 h. The lower left quadrant of the histograms shows the viable cells, which exclude PI and are negative for FITC–Annexin V binding. The lower right quadrant represents the early apoptotic cells, which are PI negative and Annexin V positive, indicating the translocation of phosphatidyl serine to the external cell surface but integrity of the cytoplasmic membrane (Vermes et al, 1995). The upper right quadrant represents the non-viable necrotic and late-stage apoptotic cells, which are positive for Annexin V binding and PI uptake. The number of apoptotic and necrotic cells increased with increasing drug concentration, and $ID_{50}$ values were calculated by plotting drug concentration against log of percentage viable cells. Figure 3 shows $ID_{50}$ values and Bcl-2/Bax ratios of treated and untreated patients.

### Correlation of Bcl-2/Bax ratios and $ID_{50}$ values

Although Bcl-2 and Bax protein expression varied within the treated and untreated groups, there was correlation between Bcl-2 and $ID_{50}$ values ($r = 0.8310$) and Bax and $ID_{50}$ values ($r = -0.6129$). When the Bcl-2/Bax ratios were correlated with chlorambucil $ID_{50}$ values, a significantly higher correlation coefficient was obtained ($r = 0.9702$). This would indicate that the combined ratio of the two proteins is more significant in determining the in vitro response to chemotherapy than the individual proteins alone ($P = 0.0001$).

**Figure 3** Chlorambucil $ID_{50}$ values and corresponding Bcl-2/Bax ratios for treated and untreated B-CLL patients. Treated patients (lanes 1–10) and untreated patients (lanes 11–22)

### DISCUSSION

Although B-CLL cells show a decreased ability to proliferate compared with normal B-cells, they have a longer lifespan in vivo, which serves to maintain the tumour cell population. In contrast, when these cells are cultured in vitro, a significant proportion of the cells die by apoptosis (Collins *et al*, 1989). Incubation with cytotoxic drugs, such as chlorambucil, accelerates this process (Begleiter *et al*, 1994). The mechanism for the induction of apoptosis by these agents is yet to be determined but it seems likely that Bcl-2 and Bax proteins play important roles in determining whether cells undergo apoptosis (Oltvai *et al*, 1993; Reed, 1995).
Dysregulation of the *Bcl-2* gene was first demonstrated in follicular lymphomas as a result of t(14, 18) translocations. Although *Bcl-2* gene rearrangements are unusual in B-CLL, high levels of Bcl-2 proteins expression have been consistently reported (Hanada *et al*, 1993; Thomas *et al*, 1996). Robertson *et al* (1996) found that 25 of 44 cases (57%) had Bcl-2 expression higher than that measured in peripheral blood lymphocytes. These present data show 14 of 22 cases (64%) with elevated Bcl-2 expression. Perhaps of more significance is the finding that 19 of 22 cases (86%) were found to express levels of Bax protein lower than those found in normal peripheral blood lymphocytes.
Previous work has indicated that B-CLL cells undergo apoptosis more readily in patients with untreated, early-stage disease (Robertson and Plunkett 1993). Recently Thomas *et al* (1996) evaluated B-CLL cells, using a semi-quantitative method, for their apoptotic response to drug treatment *in vitro* and found that cells with a high bcl-2/bax ratio were more drug resistant than cells with a low bcl-2/bax ratio. Our current data show a trend towards an apoptosis-resistant phenotype with treatment, but it remains unclear as to whether this is due to selection of a resistant population or induction of drug resistance. In addition, we have shown that Bcl-2/Bax ratios correlate with $ID_{50}$ values (as measured by in vitro apoptotic cell death) and also clinical responsiveness. Therefore, it would appear that measurement of Bcl-2/Bax ratios and in vitro apoptosis may be of prognostic value for determining whether a patient might be clinically resistant to cytotoxic chemotherapy.

### ACKNOWLEDGEMENTS

This work was supported in part by a grant from the Welsh Bone Marrow Transplant Research Fund. The authors would like to

© Cancer Research Campaign 1997

thank Dr Mike Morgan for his helpful advice on the statistical analysis.

### REFERENCES

Begleiter A, Lee K, Israels LG, Mowat MRA and Johnston JB (1994) Chlorambucil induced apoptosis in chronic lymphocytic leukemia (CLL) and its relationship to clinical efficacy. *Leukemia* **8**: s103–106

Binet JL, Auquier A, Dighiero G, Chastang C, Piguet H, Goasguen J, Vaugier G, Potron G, Colona P, Oberling F, Thomas M, Tchernia G, Jacquillat C and Boivin P (1981) A new prognostic classification of chronic lymphocytic leukemia derived from a multivariate survival analysis. *Cancer* **48**: 198–206

Cheson BD, Bennett JM, Rai KR, Grever MR, Kay NE, Schiffer CA, Oken MM, Keating MJ, Boldt DH, Kempin SJ and Foon KA (1988) Guidelines for clinical protocols for chronic lymphocytic leukemia: recommendations of the National Cancer Institute-sponsored Working Group. *Am J Hematol* **29**: 152–163

Collins RJ, Versuchuer LA, Harmon BK, Prentice RI, Pope JH and Kerr JFR (1989) Spontaneous programmed death (apoptosis) of B-chronic lymphocytic leukaemia cells following their culture *in vitro*. *Br J Haematol* **71**: 343–350

Gottardi D, Alfarano A, De Leo AM, Stacchini A, Bergui L and Caligaris-Cappio F (1995) Defective apoptosis due to bcl-2 overexpression may explain why B-CLL cells accumulate in $G_0$. *Curr Top Microbiol Immunol* **194**: 307–312

Hanada M, Delia D, Aiello A, Stadtmauer E and Reed JC (1993) bcl-2 gene hypomethylation and high level expression in B-cell chronic lymphocytic leukemia. *Blood* **82**: 1820–1828

Oltvai ZN, Milliman CL and Korsmeyer SJ (1993) bcl-2 heterodimerizes in vitro with a conserved homolog, bax, that accelerates programmed cell death. *Cell* **74**: 609–619

Pepper C, Bentley P and Hoy T (1996) Regulation of clinical chemoresistance by bcl-2 and bax oncoproteins in B-cell chronic lymphocytic leukaemia. *Br J Haematol* **95**: 513–517

Reed JC (1995) bcl-2 family proteins: regulators of chemoresistance in cancer. *Toxicol Lett* **82/83**: 155–158

Robertson LE and Plunkett W (1993) Apoptotic cell death in chronic lymphocytic leukemia. *Leuk Lymphoma* **11** (s2): 71–74

Robertson LE, Plunkett W, McConnell K, Keating MJ and McDonnell TJ (1996) Bcl-2 expression in chronic lymphocytic leukemia and its correlation with the induction of apoptosis and clinical outcome. *Leukemia* **10**: 456–459

Thomas A, El Rouby S, Reed JC, Krajewski S, Silber R, Potmesil M and Newcomb EW (1996) Drug-induced apoptosis in B-cell chronic lymphocytic leukemia: relationship between p53 gene mutation and bcl-2/bax proteins in drug resistance. *Oncogene* **12**: 1055–1062

Vermes I, Haanen C, Steffens-Nakken H and Reutelingsperger C (1995) A novel assay for apoptosis flow cytometric detection of phosphatidyl serine expression on early apoptotic cells using fluorescein labelled Annexin V. *J Immunol Methods* **184**: 39–51

© Cancer Research Campaign 1997